# *PHF21A* Related Disorder: Description of a New Case

**DOI:** 10.3390/ijms232416130

**Published:** 2022-12-17

**Authors:** Ambra Butera, Antonio Gennaro Nicotera, Gabriella Di Rosa, Sebastiano Antonino Musumeci, Girolamo Aurelio Vitello, Antonino Musumeci, Mirella Vinci, Angelo Gloria, Concetta Federico, Salvatore Saccone, Francesco Calì

**Affiliations:** 1 Department of Human Pathology of the Adult and Developmental Age, “Gaetano Barresi” University of Messina, Via Consolare Valeria 1, 98125 Messina, Italy; 2 Oasi Research Institute—IRCCS, Via Conte Ruggero 73, 94018 Troina, Italy; 3 Department Biological, Geological and Environmental Sciences, University of Catania, Via Androne 81, 95124 Catania, Italy

**Keywords:** Potocki–Shaffer syndrome, *PHF21A* gene, intellectual disability, BHC80, hypotonia, contiguous gene deletion, human chromosome 11

## Abstract

*PHF21A* (*PHD finger protein 21A*) gene, located in the short arm of chromosome 11, encodes for BHC80, a component of the Lysine Specific Demethylase 1, Corepressor of REST (LSD1-CoREST) complex. BHC80 is mainly expressed in the human fetal brain and skeletal muscle and acts as a modulator of several neuronal genes during embryogenesis. Data from literature relates *PHF21A* variants with Potocki–Shaffer Syndrome (PSS), a contiguous gene deletion disorder caused by the haploinsufficiency of *PHF21A*, *ALX4*, and *EXT2* genes. Clinical cardinal features of PSS syndrome are multiple exostoses (due to the *EXT2* involvement), biparietal foramina (due to the *ALX4* involvement), intellectual disability, and craniofacial anomalies (due to the *PHF21A* involvement). To date, to the best of our knowledge, a detailed description of *PHF21A*-related disorder clinical phenotype is not described in the literature; in fact, only 14 subjects with microdeletion frameshift or nonsense variants concerning only *PHF21A* gene have been reported. All reported cases did not present *ALX4* or *EXT2* variants, and their clinical features did not fit with PSS diagnosis. Herein, by using Exome sequencing, and Sanger sequencing of the region of interest, we describe a case of a child with a paternally inherited (mosaicism of 5%) truncating variant of the *PHF21A* gene (c.649_650del; p.Gln217ValfsTer6), and discuss the new evidence. In conclusion, these patients showed varied clinical expressions, mainly including the presence of intellectual disability, epilepsy, hypotonia, and dysmorphic features. Our study contributes to describing the genotype–phenotype spectrum of patients with PHF21A-related disorder; however, the limited data in the literature have been unable to provide a precise diagnostic protocol for patients with *PHF21A*-related disorder.

## 1. Introduction

The *PHF21A* (*PHD finger protein 21A* gene, OMIM*608325) encodes BHC80, a component of a BRAF35/histone deacetylase (HDAC) complex (BHC) that mediates repression of neuron-specific genes through the cis-regulatory element known as repressor element-1 (RE1) or neural restrictive silencer (NRS) [1]. It is involved in the regulation of neuronal stem cell fate and neuronal gene transcription and modulation [2,3,4,5,6]. BHC80 is mainly expressed in the human fetal brain and skeletal muscle and acts as a modulator of several neuronal genes during embryogenesis [6,7,8,9,10]. Specifically, BHC is recruited at repressor element 1 or neuron-restrictive silencer element (RE1/NRSE) sites by RE1 silencing transcription factor (REST) and deacetylates and demethylates specific sites on histones, thereby acting as a chromatin modifier [3]. The ancestral presence of *PHF21A* emphasizes a crucial role in early human development and led to hypothesize a role in autism, attention deficit hyperactivity disorder (ADHD), and epilepsy onset [11]. Moreover, Kim et al. [6] suggested a contribution of *PHF21A* in hypotonia phenotypes traceable to its abundance in skeletal muscle [6].

*PHF21A*, jointly with *EXT2* (*exostosin glycosyltransferase 2*, OMIM*60821) and *ALX4* (*Aristaless-like homeobox 4*, OMIM*605420), is considered one of the three genes involved in Potocki–Shaffer Syndrome (PSS, MIM: 601224). PSS is a contiguous gene deletion disorder caused by the haploinsufficiency of multiple functionally unrelated genes on the proximal short arm of chromosome 11, specifically in the 11p12-p11.2 region [6,7,12]. In particular, the involvement of *PHF21A* is considered causative of intellectual disability (ID) and craniofacial anomalies, whereas *EXT2* is associated with multiple exostoses, and *ALX4* with parietal foramina [6,7,11,13,14,15]. The concomitant presence of all these clinical features makes the diagnosis of PSS very difficult [6,9].

However, the diagnosis of PSS can be made even without all the above signs or symptoms. Genotype–phenotype correlation studies defined a 2.1 Mb critical region in PSS patients that spans from D11S1393 to D11S1385/D11S1319 [15], where a basic gene for cognitive development and/or cognitive function likely resides around 45.6–46.7 MB from the 11p terminus [15,16]. In line with these data, several authors agree that haploinsufficiency of *PHF21A* is probably sufficient to produce the ID observed in PSS patients and hypothesized a causative synergic role of several genes included in the microdeletion [13,16]. Accordingly, also described was a PSS patient without ID whose deletion involving the 11p11.2-p12 region does not encompass *PHF21A* gene [17]. It should be also stressed that, in a zebrafish model, a *PHF21A* orthologous suppression produced abnormalities in the development of the head, face, and jaw, demonstrating an etiologic role of *PHF21A* in many facial dimorphisms (i.e., brachy/microcephaly and mild micrognathia) [18].

To date, to the best of our knowledge, a detailed description of *PHF21A*-related disorder clinical phenotype is not reported in the literature; in fact, only 14 subjects with microdeletion frameshift or nonsense variants concerning only *PHF21A* gene have been described. Thus, new subjects carrying mutations in the *PHF21A* gene represent a relevant finding to improve our knowledge of PSS disease. Herein, we report the case of a Caucasian patient in which a paternally inherited (who presented a 5% mosaicism) truncating variant (c.649_650del; p.Gln217ValfsTer6) in the 11p11.2 region was identified, involving the *PHF21A* gene.

## 2. Case Presentation

### 2.1. Patient

The patient is a boy 16 years old (yo) of Caucasian origin. He is the first child of unrelated healthy parents, and he has a younger brother affected by a specific learning disorder. He was born full term after a normal pregnancy with a birth weight of 3150 g (15th–50th centile). He presented a global developmental delay: he walked without support at 22 months and could pronounce simple words at 12 months. At three yo, a speech delay was detected and confirmed at 5 yo (normal bilateral audiological profile). At the age of 11 yo, he underwent an adenoidectomy. Additionally, he presented sleep disturbances (chronic nocturnal snoring) and frequent diurnal asthenia.

**Figure 1:**
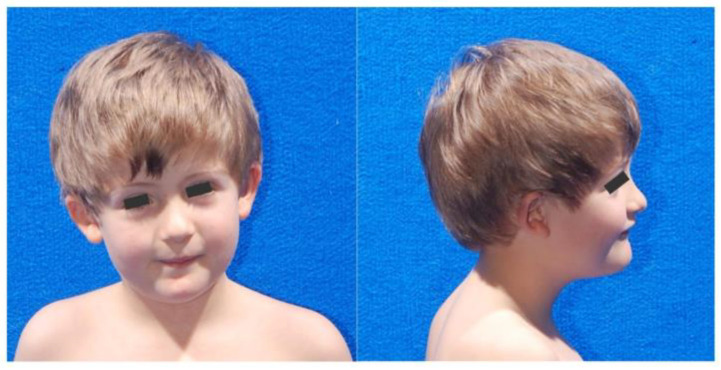
Pictures of the patient presenting a truncating variant (c.649_650del; p.Gln217ValfsTer6) of the *PHF21A* gene. **Left**: front picture showing round face and reduced head circumference. **Right**: profile picture showing moderate micrognathia and low-set, anteriorly rotated ears.

At the age of 13 years, he presented his first seizure, characterized by a sudden loss of muscle tone, loss of consciousness, and followed by vomiting. Electroencephalogram (EEG) showed multifocal paroxysmal discharges, occasionally preceded by burst suppression. His growth parameters at this age were within normal limits. Head circumference was 50 cm (<3rd centile). Physical examination (Figure 1) showed no skull defects, although he presented moderate micrognathia and small, anteriorly rotated ears. Moreover, he had mild hypotonia, modest joint laxity, left thoracic scoliosis, knocked knees, and flat feet. He presented fine- and gross-motor difficulties and mild dysmetria on the index-to-nose test. The language was characterized by phonetic errors and limited semantic–pragmatic abilities.

Additionally, he showed anxiety and emotional immaturity. Abdomen ultrasound showed echogenic spots (“urinary sand”) bilaterally expressed. Electrocardiogram, routine blood tests, and brain magnetic resonance imaging (MRI) were not informative. A second EEG, performed in sleep, at the same age, did not show anomalies. Electromyography (EMG) showed small amplitude polyphasic myopathic potential, with rapid recruitment, in the proximal and distal areas of arms and legs. Mandibular prognathism class III and consequential malocclusion pattern were detected on a cranial computed tomograph (CT). Ophthalmologic valuation detected myopic astigmatism.

### 2.2. Genetic Data

**Figure 2:**
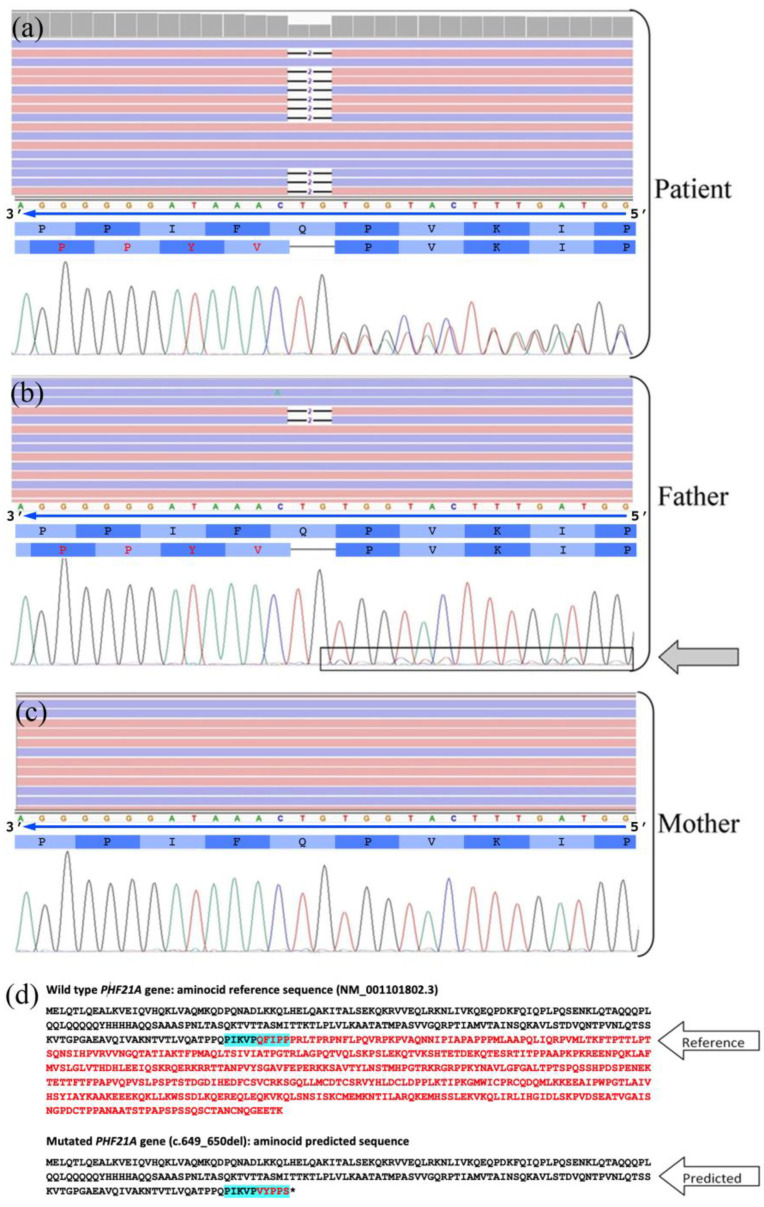
Visualization of the frameshift mutation in *PHF21A* gene: (**a**–**c**) from top to bottom (i) the NGS data with Integrative Genomics Viewer, (ii) the nucleotide sequence with the direction of the reading frame, (iii) the wild-type and the mutated aminocid sequences, and (iv) the electropherogram obtained by Sanger sequencing. The two-bases deletion c.649_650del (p.Gln217ValfsTer6) determining the frameshift mutation in *PHF21A* gene (NM_001101802) is represented in the colored lines of the NGS viewer with or without deletion (some reads are shown as examples). In the mutated protein, the divergent aminoacids were indicated in red. The heterozygous patient shows 50% reads with the causative deletion, whereas the father presents the heterozygous mutation in ~5% of his blood cells (7 of 146 reads), and the mother only the homozygous normal *PHF21A* allele. It is evident in the Sanger sequences the difference among patient, father, and mother with the deletion determining the phase displacement of the two allele sequences highlighted in the right part of the electropherograms in the patient and in the father. (**d**) Aminocid sequence of the PHF21A protein in the wild-type condition, and in the mutated form following the two-bases deletion; five aminoacids were translated, and then the protein was truncated due to the presence of a STOP codon (p.Gln217ValfsTer6). The aminoacid sequence shown in the upper panels is highlighted in pale blue.

Array-CGH analyses 60k did not reveal chromosomal imbalances. Nuclear DNA was isolated from our subject and his parents. Clinical exome sequencing (CES) revealed a heterozygous frameshift mutation in the *PHF21A* gene (c.649_650del; p.Gln217ValfsTer6). This mutation variant causes an early stop codon after incorporating six missense aminoacids, resulting in a truncated protein. The mutation was not found in gnomAD exomes and genomes samples. PhyloP100way scores (9.075) based on multiple alignments of 99 vertebrate genome sequences to the human genome indicate a more conserved site. ACMG Classification: pathogenic. This variant had a heterozygous transmission from the phenotypically normal father, who presented a 5% mosaicism of the *PHF21A* gene. Its causative role was confirmed by Sanger sequencing. The comparison between the normal maternal *PHF21A* gene electropherogram and the evidence of the 5% mosaicism in the paternal one corroborates our evidence (Figure 2).

### 2.3. Materials and Methods

#### 2.3.1. DNA Preparation

Written informed consent was obtained from the parents of the patient. The study was conducted in accordance with the Declaration of Helsinki. Nuclear DNA from the patient and his parents was isolated from peripheral blood leucocytes using the standard salt phenol–chloroform extraction method.

#### 2.3.2. Array-CGH and NGS Sequencing

An array-CGH (resolution 400 k) was performed. Genomic DNA was used for DNA library preparation (TRIOS) and exome enrichment using the Agilent SureSelect V7 kit according to manufacturer instructions. A sequencing run was performed on an Illumina HiSeq 3000 instrument. This approach achieved 97% of regions covered at least 20x. Data analysis has been performed using an analysis pipeline based on public tools. We filtered the identified variants according to: (i) recessive/de novo/X-linked pattern of inheritance, (ii) allele frequencies (mean average frequency, MAF) <1% using as reference the following genomic datasets: 1000 Genomes, ESP6500, ExAC, gnomAD, as previously described [19]. In silico analyses were performed by Varsome (ACMG criteria). To confirm the identified mutation, Sanger sequencing was performed using the BigDye Terminator v1.1 Cycle Sequencing Kit (Life Technologies, CA, USA) with an ABI 3130 instrument (Life Technologies, CA, USA).

## 3. Discussion

Herein, we report a new patient carrying a paternally inherited (mosaicism of 5%) truncating variant (c.649_650del; p.Gln217ValfsTer6) in the 11p11.2 region involving the *PHF21A* gene, detected by CES trio analysis. The product of *PHF21A* is a component of BHC, and it is involved in the regulation of neuronal stem cell fate and neuronal gene transcription and modulation [3].

In 2007, Lan et al. firstly showed that a *PHF21A* loss leads to the de-repression of *REST* target genes in non-neuronal cells [20]. Subsequently, other researchers hypothesized that a large number of histone methylation regulating genes represent a possible genetic basis for neurodevelopmental disorders, such as ID and autism [21,22]. In line with these data, mutations of histone-modifying enzymes, such as histone methyltransferase and demethylases, in syndromic ID were clearly reported [20]. Despite this, little is known about the molecular mechanisms behind histone methylation dynamics and their contribution to neurodevelopmental patterns.

**Figure 3:**
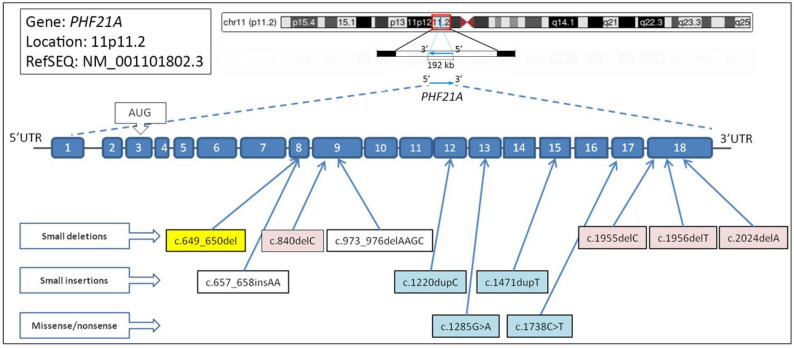
*PHF21A* gene organization, and major described mutations. Human *PHF21A* gene is located in chromosome 11 (band p.11.2). The gene, composed by 18 exons, is transcribed from its centromeric to telomeric side (upper part of the figure). At the bottom part of the figure are shown the previously reported major mutations in *PHF21A* gene, in male (blue) and female (pink). The mutation found in our patient is highlighted in yellow. The identified deletions, duplications, and point mutations in the coding sequence are depicted below the exons.

To date, conspicuous mutations involving the short arm of chromosome 11 have been reported as causative of PSS, a complex genetic syndrome characterized by ID, craniofacial anomalies, multiple exostoses, and biparietal foramina. *PHF21A* is one of the genes included in this region (Figure 3); however, only 14 subjects with microdeletions, frameshift or nonsense variants involving exclusively the *PHF21A* gene have been reported in the literature (see Table 1) [6,11,22,23,24,25,26]. All of the reported variants lead to a truncating protein, hence altered and not fully functioning. Twelve of the mentioned patients carry a de novo *PHF21A* pathogenic variant, whereas two have no data regarding the inheritance [6,23,24].

None of the 15 patients (including our patient) reported exostoses or biparietal foramina, showing instead variable clinical features partially overlapping with those typical of PSS. In addition, Ung and et al. [26] reported the case of a patient affected by diabetic retinopathy with hyperglycemia-induced suppression of *PHF21A* without providing further information.

With the exception of three reported cases in which a complete description of the phenotype is lacking, developmental delay and ID are common features for patients with PHF21A-related disorder. Overall, patients showed a large spectrum of clinical features, from global delayed psychomotor development (with language delay) to a severe ID. Moreover, sometimes ID may be associated with verbal and visual–constructive dyspraxia, difficulties related to abstraction, and ADHD, as comorbidity disorders [6].

*PHF21A* gene is also known as causative of the craniofacial anomalies described in PSS. In line with these data, macrocephaly has been described in four subjects, two patients showed plagiocephaly, and another showed hyperplasia of the mid-face and triangular face, whereas our patient presented microcephaly. All clinical features are summarized in Table 1.

It is interesting to note that, firstly, Kim et al. [18] and then Labonne et al. [13] sustained an etiologic role of *PHF21A* haploinsufficiency in facial dysmorphism insurgence observed in PSS patients. Despite this, McCool et al. [17] reported a patient with distinct craniofacial abnormalities and without *PHF21A* deletion, suggesting the role of further causative factors in their occurrence.

Other dysmorphisms regarding hands and feet fingers were detected in a large number of subjects (6/15). Tapering fingers were reported in four subjects, two patients presented with syndactyly, and four patients had clinodactyly. Our patient did not show significant finger dysmorphisms.

As previously reported by Kim et al. [6], there may be a correlation between *PHF21A* variants and musculoskeletal involvement. In fact, muscular tissue is the second district in which the *PHF21A* gene is expressed. Therefore, truncating protein occurrence may be causative of hypotonia and motor impairment. Indeed, impaired motor skills were detected in eleven patients, most of whom (7/11) also presented hypotonia. Our patient only showed hypotonia without any other significant involvement of the motor domain.

Interestingly, our patient showed EMG alterations. In particular, he presented a myopathic EMG pattern. It is worthy to note that none of the reported patients underwent EMG. Conversely, we support the investigation of striated skeletal muscle functionality in this cluster of patients, as the involvement of the striated skeletal muscle in the PSS could not be evaluated or underestimated.

Literature data, including our patient, also showed that 7/15 reported cases with *PHF21A* variants suffered from epilepsy. The affected subjects showed heterogeneous seizure patterns, such as flexor spasms, complex partial seizures, repeated eye blinking and eye-rolling seizures, and generalized tonic–clonic seizures [6,11,23]. Compared with other genes related to drug-resistant epileptic syndromes, none of the reported cases with *PHF21A* variants showed an epileptic encephalopathy [27,28,29].

Our patient presented with a single generalized seizure at the age of 13 years. He did not present other seizures; therefore, no pharmacologic therapy was needed. Similarly, no reported cases showed a peculiar EEG pattern or pathognomonic brain MRI features.

To date, *PHF21A* variants were also described in association with behavioral problems. Unfortunately, specific data about behavioral problems, ADHD, or personality disorders are not available for these subjects. The limited data available in the literature highlight a moderate incidence of ADHD and anxiety disturbances in *PHF21A* patients. In this regard, six subjects suffered from ADHD. Meanwhile, five patients, including ours, experienced various anxious traits. Our patient also revealed emotional immaturity. Moreover, Kim and et al. [6] previously suggested a possible role of *PHF21A* in autism expression; as evidence of this, 6 patients of the 14 previously reported fall within the category of autism spectrum disorder; however, for two of them, there are no other details available [6,22,24].

Finally, five subjects were also affected by obesity. Even if our patient’s body mass index falls within the normal range, he showed a significant abdominal distribution.

Concerning genetic variants, worthy of mention is that our patient is the only one with an inherited deletion. Specifically, it is a paternally inherited truncating variant in *PHF21A* gene. However, this variant had a heterozygous transmission from the phenotypically normal father, who presented an exiguous mosaicism percentage (5%) of *PHF21A*. It is current practice in genetic counseling to reassure parents when a certain de novo mutation is found.

Identifying mosaicisms present technical difficulties, especially when the percentage is very low. Given our result, a more cautious approach in counseling would be advisable in all cases with an apparently de novo mutation.

## 4. Conclusions

The syndromic picture specifically related to *PHF21A* mutations is still poorly described in the literature; in fact, only 15 cases (including our patient) have been reported. These patients showed various clinical features, mainly including the presence of ID, epilepsy, hypotonia, and dysmorphic traits.

Identifying patients with syndrome related to the 11p11.2 region could be challenging due to the heterogeneous clinical phenotype presented by the affected children. This is even more valid for the patients affected only by *PHF21A* disease-causative variants. Moreover, it should be important to improve the clinical-diagnostic protocol of these patients, and it may be useful to add the *PHF21A* gene to NGS ID or epilepsy panel. In addition, considering that the *PHF21A* gene is abundantly expressed in skeletal muscles, over half of patients presented hypotonia, and, firstly, our patient showed a myopathic EMG pattern; the EMG analysis should be included in the clinical–diagnostic protocol of these patients.

To date, the few data available in the literature did not consent us to define a specific diagnostic protocol for patients with *PHF21A* variants. Hence, further studies are needed to better understand and explain the available information on this topic. Otherwise, it should be noted that our study contributes to enrich the genotype–phenotype spectrum of PHF21A-related disorders. Furthermore, the paternal mosaicism described in our report highlights the necessity of familial genetic counseling, which can provide more accurate data regarding the risk of transmission on further conceiving.

## Figures and Tables

**Table 1:** Summary of the present patient clinical features, compared to the other 14 reported patients.

	Pt. 1	Pt. 2	Pt. 3	Pt. 4	Pt. 5	Pt. 6	Pt. 7	Pt. 8	Pt. 9	Pt. 10	Pt. 11	Pt. 12	Pt. 13	Pt. 14	Pt. 15
Age (years)	16	13	3	9	10	18	6	18	N/A	N/A	N/A	N/A	9	3	26
Sex	Male	Female	Male	Female	Male	Male	Male	Female	Female	N/A	Female	N/A	Male	Male	Female
Nucleotide change	c.649_650 del	c.1955 delC	c.1285 G > A	c.1956 delT	c.1738 C > T	c.1471 dupT	c.1738 C > T	c.2024 delA	c.840 delC	c.976_979 delAAGC	c.1153 delA	c.1545 delA	c.657_658 insAA	c.1220 dupC	c.1171 A > T
Effect on protein	p.Q217V fsTer6	p.P652L fsX104	p.G429S	p.P652P fsX104	p.R580Ter	p.C491L fsX81	p.R580Ter	p.Q675R fsX81	p.I281S fs*14	p.K326fs	p.S385A fs*30	p.E517K fsTer14	p.P220Nfs*48	p.E408R fs*3	p.K391Ter
Inheritance ^(b)^	P.I. (5%)	D.N.	D.N.	D.N.	D.N.	N/A ^(c)^	D.N.	D.N.	N/A	D.N.	D.N.	D.N.	D.N.	D.N.	D.N.
Developmental delay	+	+	+	+	+	+	+	+	N/A	+	N/A	N/A	+	+	+
Intellectual disability	+	+	+	+	+	+	+	+	N/A	+	N/A	N/A	+	+	+
Facial dysmorphism	+	+	+	+	+	−	+	+	N/A	+	N/A	N/A	+	+	+
Cranial anomalies ^(d)^	Mic	Mac	−	−	Plc	−	−	Mac	N/A	+ ^(e)^	N/A	N/A	Mac	Mac	Plc
Autism	−	+	N/A	−	−	+	−	+	+	N/A	+	N/A	−	N/A	−
Epilepsy/seizures/spasms	+	+	+	+	+	−	−	−	−	−	−	−	−	+	+
Language delay	+	+	+	+	+	+	+	+	N/A	+	N/A	N/A	+	+	+
Tapering fingers	−	+	+	+	−	−	−	−	N/A	N/A	N/A	N/A	−	+	−
Clinodactily	−	+	+	+	−	+	−	−	N/A	N/A	N/A	N/A	N/A	N/A	−
Syndactily	−	−	+	−	+	−	−	−	N/A	N/A	N/A	N/A	N/A	N/A	−
Impaired motor skills	−	+	+	+	+	+	+	+	N/A	+	N/A	N/A	+	+	+
Hypotonia	+	+	+	+	N/A	−	−	−	N/A	N/A	N/A	N/A	+	+	+
ADHD	−	+	N/A	+	+	+	−	N/A	N/A	N/A	N/A	N/A	+	N/A	+
Anxiety disorder	+	+	N/A	+	N/A	−	+	+	N/A	N/A	N/A	N/A	N/A	N/A	−
Neurobehavioral problems	−	+	+	+	+	+	−	+	N/A	+	N/A	N/A	N/A	N/A	+
Obesity	−	+	−	+	−	+	−	+	N/A	N/A	N/A	N/A	N/A	N/A	+
Reference	^(a)^	[6]	[6]	[6]	[6]	[6]	[6]	[6]	[23]	[24]	[21]	[25]	[11]	[11]	[22]

^(a)^ Our patients described in the present study. Pt.: patient. N/A: not available. ^(b)^ MiC: microcephaly; MaC: macrocephaly; PlC: plagiocephaly. ^(c)^ Hyperplasia of midface and triangular face. ^(d)^ P.I.: paternally inherited; D.N.: de novo. ^(e)^ Not found in mother.

## Data Availability

Data supporting the findings of this study are available from the corresponding authors on request.
